# Doxycycline-Associated Ulcerative Esophagitis: A Report of a Rare Case

**DOI:** 10.7759/cureus.58079

**Published:** 2024-04-11

**Authors:** Kunal Priyadarshi, Suman Panda, Amiya Ranjan, Dheeraj Yadav, Zaid Nafe

**Affiliations:** 1 Internal Medicine, Manipal Tata Medical College, Jamshedpur, IND; 2 General Medicine, Tata Main Hospital, Jamshedpur, IND; 3 Internal Medicine and General Medicine, Tata Main Hospital, Jamshedpur, IND; 4 Gastroenterology, Tata Main Hospital, Jamshedpur, IND; 5 Community Medicine, Dr. Vaishampayan Memorial Government Medical College (VMGMC), Solapur, IND

**Keywords:** antibiotics, tetracycline, endoscopic evaluation, doxycycline, medication-induced esophageal injury

## Abstract

This study addresses the risks of medication-induced esophageal injury through a case presentation of a 30-year-old patient treated with doxycycline. The case highlights the importance of proper medication administration and the role of endoscopic evaluation in diagnosis and management. The broader discussion emphasizes the prevalence of such injuries, especially with antibiotics, and factors influencing their occurrence. A clinical study illustrates the corrosive effect of tetracycline, highlighting the role of pH and hyperosmolar properties. The study concludes with a reminder of the critical role of healthcare professionals in recognizing and managing medication-induced esophagitis, with endoscopy as a key diagnostic tool.

## Introduction

Medication poses a considerable risk to the esophageal mucosa, through either direct mucosal damage or systemic effects. Determining the precise incidence and prevalence of pill-induced esophagitis is challenging due to underreporting, with most cases going unreported and medical attention being sought primarily for severe instances. Numerous medications have been identified as potential contributors to esophagitis, and various risk factors, including altered anatomy or motility, patient positioning, medication size, and the volume of fluid consumed during administration, have been extensively documented [1]. Over the past four decades, more than 650 instances of esophagitis attributed to 30 different drugs have been documented [2]. Among these drugs, half of the reported cases involve tetracycline, doxycycline, and clindamycin [3]. Esophageal injuries are significantly more prevalent with capsule forms than tablet forms, as capsules can readily adhere to the esophageal mucosa [4]. Clinical manifestations include intense retrosternal pain and dysphagia, typically manifesting hours or days after drug ingestion [3]. We are going to discuss a case involving a patient who experienced pronounced heartburn and difficulty swallowing following the consumption of doxycycline.

## Case presentation

A 30-year-old man without any pre-existing health conditions was receiving treatment for periorificial dermatitis, following a regimen of doxycycline at a dosage of 100 mg twice a day. On the fifth day of the treatment, the patient experienced intense retrosternal pain and odynophagia upon waking up. On that particular day, he faced difficulty consuming solid foods and continued to experience significant heartburn throughout, despite taking medications for symptom relief.

The following day, the patient sought medical help and disclosed that he had missed the night dose of his medication. He mentioned waking up in the middle of the night and taking the medication while lying down without consuming any fluids. Despite this, the symptoms persisted the next morning, prompting the patient to seek medical attention. Upon admission, he underwent fasting, underwent laboratory tests, received a prescription for a proton pump inhibitor, and was scheduled for an upper gastrointestinal endoscopy.

**Figure 1:**
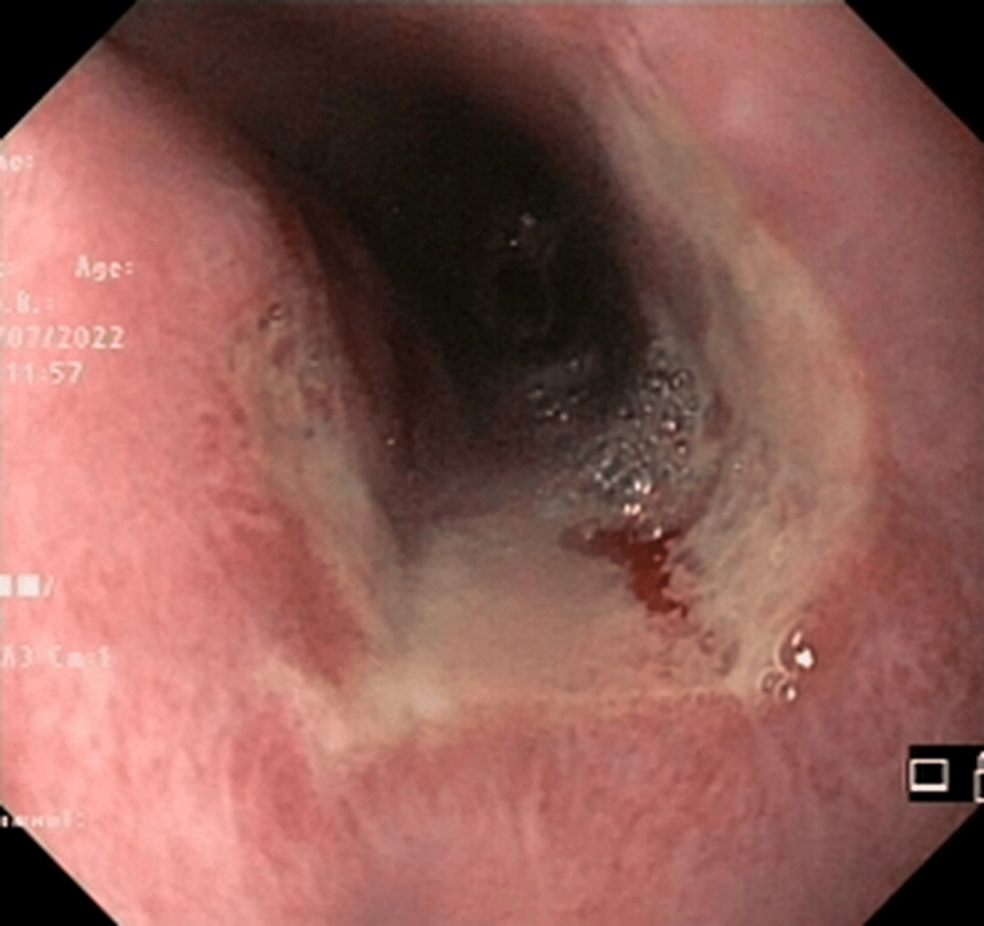
Ulcer spanning from 25 cm to 31 cm from the oral cavity

No irregularities were observed in the laboratory results, and the upper gastrointestinal endoscopy unveiled a substantial ulcer spanning from 25 cm to 31 cm from the oral cavity (Figure 1).

**Figure 2:**
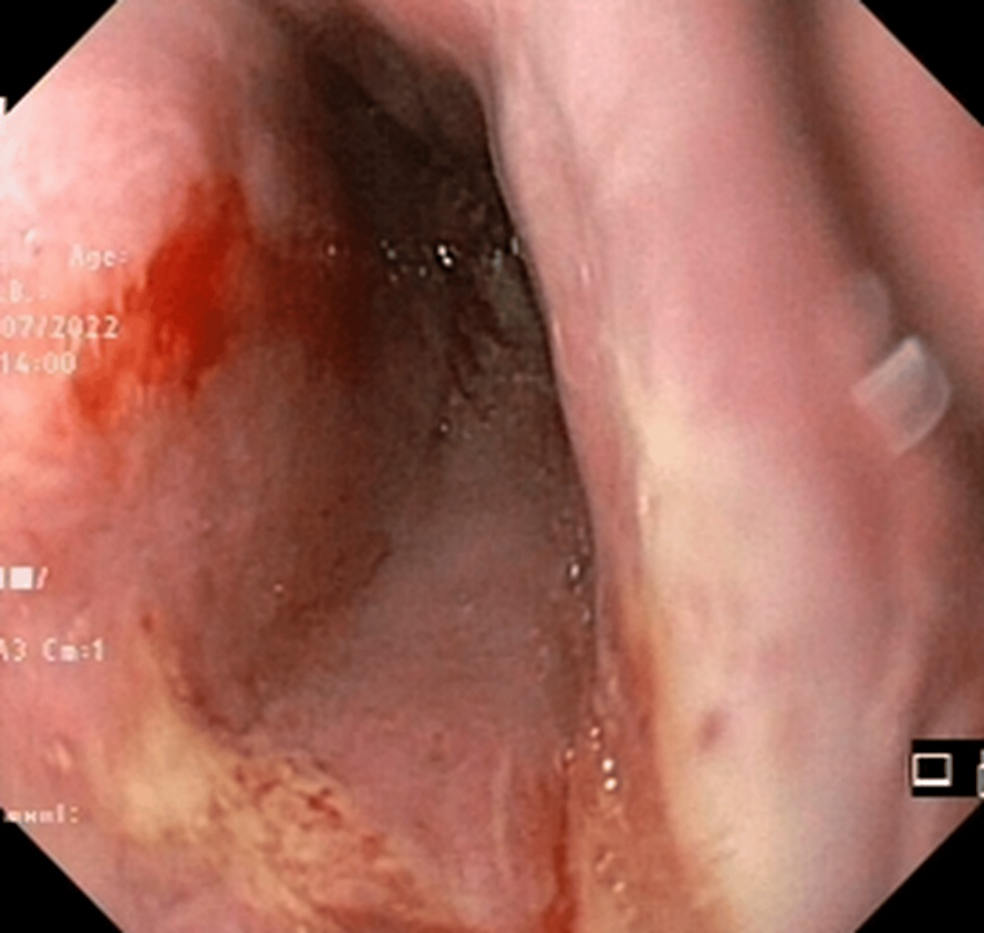
Mucosal fragility upon scope passage

The ulcer showed no signs of active bleeding but displayed fragility upon scope passage (Figure 2).

The procedure did not reveal any other anomalies, and biopsies were not conducted on the ulcer due to its known cause.

Following the procedure, a liquid diet was introduced and well-tolerated. The subsequent day saw a notable enhancement in symptoms, with the patient experiencing no pain, nausea, or vomiting despite dietary intake. The individual was released from the hospital and prescribed a proton pump inhibitor and sucralfate, and a clinical follow-up was arranged for one month later. During the follow-up, the patient remained asymptomatic, with no reports of pain or dysphagia. The diet advanced to solid foods by the second week, and the medication was ceased three days before the scheduled appointment without any complications. Given the complete resolution of symptoms despite the dietary progression and cessation of medication, no further treatment or additional endoscopic procedures were deemed necessary at this stage.

## Discussion

In our contemporary society heavily reliant on medications, the seemingly routine act of taking pills is commonly perceived as harmless. Nevertheless, over 200 documented cases of medication-induced esophageal injury involve around 26 different types of medications [5]. The actual prevalence of this issue remains uncertain. According to a 1978 Swedish study, an incidence of 3.9 cases per 100,000 population was reported [3]. Females are affected more frequently than males. Typically, individuals take a dose of medication at bedtime with minimal water, immediately lying down to sleep. Within a few hours to days, symptoms such as odynophagia or persistent chest pain, exacerbated by eating, may manifest. The transportation of medication into the stomach is often delayed in individuals with pre-existing esophageal or cardiac conditions. Antibiotics, particularly tetracycline or doxycycline capsules, are frequently implicated in medication-induced esophagitis. Other common culprits include slow-release potassium chloride and quinidine [5].

The esophageal mucosal injury can be attributed to local acid burn and hyperosmolar properties of certain medications. A study involving 40 patients who were prescribed tetracyclines and subsequently developed esophageal ulcers found that their endoscopic appearance was similar. Additionally, an experimental part of the study demonstrated the corrosive effect of tetracycline on cat esophagi [6]. Drugs with low pH, such as doxycycline, ferrous sulfate, and ascorbic acid, can also induce esophagitis through a similar mechanism. There are reports indicating that potassium chloride-induced esophagitis results from local tissue destruction and vascular injury due to its hyperosmolar properties [7]. Sustained-release formulations pose a higher risk of causing esophagitis compared to other preparations. Similarly, gelatin capsules, being hygroscopic, are more prone to causing esophagitis. They may adhere to the esophageal wall due to either mechanical pressure or chemical injury upon drug release [8].

The symptoms encompass retrosternal pain or heartburn, odynophagia, dysphagia, and a documented history of consuming medications recognized for causing esophageal injury [9]. Typically, the onset of these symptoms transpires within the initial three days, frequently emerging within the initial hours following the ingestion of the implicated drug. Patients frequently recount swallowing a pill without water, often at bedtime [9]. Symptoms typically subside within the initial week following appropriate evaluation, although complications such as perforation and strictures have been previously documented.

Thus, seeking medical consultation and undergoing an endoscopic assessment are imperative for a thorough diagnosis and appropriate treatment recommendations. The subsequent course of action in managing the condition involves discontinuing the causative medication and initiating acid suppression therapy.

## Conclusions

This case underscores the risks of medication-induced esophageal injury, emphasizing the need for medical awareness. The patient's experience with doxycycline-induced esophagitis highlights the importance of proper medication administration. Endoscopic evaluation played a crucial role in diagnosing and guiding the management plan. The broader discussion emphasizes the underreported nature of medication-induced esophagitis, with antibiotics as common culprits. Factors such as medication properties and patient behaviors significantly influence these injuries. This case emphasizes the critical role of medical professionals in recognizing and managing such cases, with endoscopy serving as a valuable diagnostic tool. Healthcare practitioners should be vigilant about potential adverse effects on the esophagus, especially when symptoms arise post-medication ingestion.
